# Epidemiological trends of head and neck Cancer survivors in Alberta: towards improved understanding of the burden of disease

**DOI:** 10.1186/s40463-020-00443-4

**Published:** 2020-07-06

**Authors:** Jin Soo Song, Patrick Vallance, Vincent Biron, Caroline C. Jeffery

**Affiliations:** 1 Faculty of Medicine and Dentistry, University of Alberta, Edmonton, Canada; 2 Division of Otolaryngology – Head & Neck Surgery, Department of Surgery, University of Alberta, 1E4 Walter Mackenzie Centre, 8440-112 Street NW, Edmonton, Alberta T6G 2B7 Canada

**Keywords:** Demographics, Epidemiology, Head and neck Cancer, Survivors, Survivorship, Canada, Alberta

## Abstract

**Background:**

With an increase in the incidence of human papillomavirus (HPV)-associated oropharyngeal squamous cell carcinoma (OPSCC) and more favourable survival outcomes, there is now a population of head and neck cancer survivors that are different from preceding decades. In addition, their long-term survivorship issues have become increasing research interests. This study was undertaken to determine the changing epidemiological trends of head and neck cancer survivors in Alberta to better anticipate future demands on healthcare services.

**Methods:**

The Alberta Cancer Registry was queried for adult (aged > 18 years), head and neck cancer (HNC) patients who were at least 1-year post-treatment completion between 1997 to 2016. Cutaneous head and neck and thyroid cancer patients were excluded. Extracted data was then used to calculate the incidence and prevalence of early (< 5 years from treatment), intermediate (5 to < 10 years from treatment), and late (> 10 years from treatment) survivors of head and neck cancer. Point prevalence of HNC survivors in 2005, 2010, and 2015 were then further stratified by gender, sub-site and age.

**Results:**

Over this time period, head and neck cancer survivors tended to be younger (64.0 vs. 62.1, *p* = 0.046) and male (M:F 2.45:1 vs 2.54:1). In 1997, the predominant subsites were the oral cavity and larynx at 45.8% and 30.9%, respectively. In 2015 the predominant subsites were the oral cavity and oropharynx at 33.0% and 29.4%, respectively. Within the cohort, the prevalence of late HNC survivors increased to 13.3 per 100,000 people in 2015.

**Conclusions:**

There is a significant population of head and neck survivors who are younger, male, and more than 10 years post-treatment. While oral cavity cancers have shown stable disease prevalence in recent decades, the number of OPSCC survivors have increased. With an improved understanding of the distribution and characteristics of HNC survivors, a more guided healthcare support network can be fostered for these patients.

## Background

Changes in the incidence and demographics of new head and neck cancer (HNC) diagnoses are well documented [1, 2]. Historically, oral cavity squamous cell carcinomas (OCSCC) have comprised the largest proportion of HNCs [3]. In western countries, previously higher rates of oral cavity and laryngeal malignancies have decreased in response to a decreasing prevalence of smoking [1, 3, 4]. Conversely, oropharyngeal squamous cell carcinomas (OPSCC) most commonly presenting on the base of tongue and palatine tonsils have demonstrated a dramatic increase in incidence [2, 4]. Patients diagnosed with OPSCC do not necessarily have classic lifestyle risk factors of chronic cigarette smoking and alcohol consumption, as the majority of cases are associated with human papillomavirus (HPV) [2, 4]. Fortunately patients with HPV-associated OPSCC generally have more favourable survival outcomes when compared to equivalently staged HPV negative cohorts [2].

The overall survivorship within the HNC population has traditionally been poor. Fortunately, this too has changed with the use of evidence-based treatment paradigms and advances in adjuvant and treatment options, resulting in improved oncologic outcomes [1, 5]. The aforementioned HPV associated OPSCC cohort also plays a large role in HNC survivorship, as largescale studies cite 5-year survival rates of 90% [6]. A 2017 report from the Canadian Cancer Society details a 40% decrease in age standardized mortality in men, who compromise an estimated 70% of new HNC patients [7]. As the long-term survival outcomes continue to improve in HNC patients, there is a responsive shift in focus to understanding and managing the long-term treatment sequelae and morbidities, includes impacts on speech, swallowing, social interactions and quality of life [8, 9].

The objective of our study was to accurately determine the epidemiological trends of HNC survivors in Alberta to inform planning of healthcare services and address the health needs of this population.

## Methods

Approval through the Health Research Ethics Board of Alberta – Cancer Committee (HREBA.CC-17-0210) was obtained prior to study commencement. Patients were identified through the Alberta Cancer Registry (ACR), a prospectively collected population-based registry containing data from patients treated in Alberta at 2 tertiary cancer centres and one associated cancer centre; patients include residents of Alberta, northern British Columbia, Saskatchewan, and the North West Territories. The ACR is funded and maintained by Alberta Health Services Cancer Care and has a sophisticated system of patient registration, coding, quality assurance reviews, and case ascertainment with known high accuracy for the head and neck cancer population [10]. Inclusion criteria included all adult patients aged 18 years or older, with a diagnosis of mucosal and salivary HNC and underwent curative intent treatment between 1997 and 2015. Exclusion criteria included cutaneous and thyroid malignancies, patients who did not undergo or complete treatment, or were missing demographic or follow up information. Duplicate patients were also removed (i.e. patients with secondary primaries in the head and neck).

To evaluate the annual incidence of new head and neck cancer survivors, we counted the number of individuals who were alive and free of disease and whose entered one-year post-treatment during the calendar year evaluated. Patients were then stratified by age, subsite, and sex. The point prevalence of early, mid-, and late survivors, was further analyzed by examining the 2000 to 2015 time-frame. This period was partitioned into three specific time points of 2005, 2010 and 2015. Alive patients at each time point were stratified into early (< 5 years from diagnosis), intermediate (5 to 10 years from diagnosis) and late (> 10 years from diagnosis) subgroups by calculating the difference between date of last known alive status and date of diagnosis to generate the years alive since diagnosis. The date from diagnosis was chosen since this date was most reliably reported in the registry. Point prevalence per 100,000 people at each time point was calculated based on the total count of appropriate cases divided by the reported fourth quarter Alberta population size reported by the Government of Alberta for the specified year multiplied by 100,000 [11]. The distribution of survivors at each time point was then stratified by gender and subsite.

Excel 2018 (Microsoft Corporation, WA, USA) was used to tabulate counts, proportions, and perform t-test parametric comparisons.

## Results

**Fig. 1:**
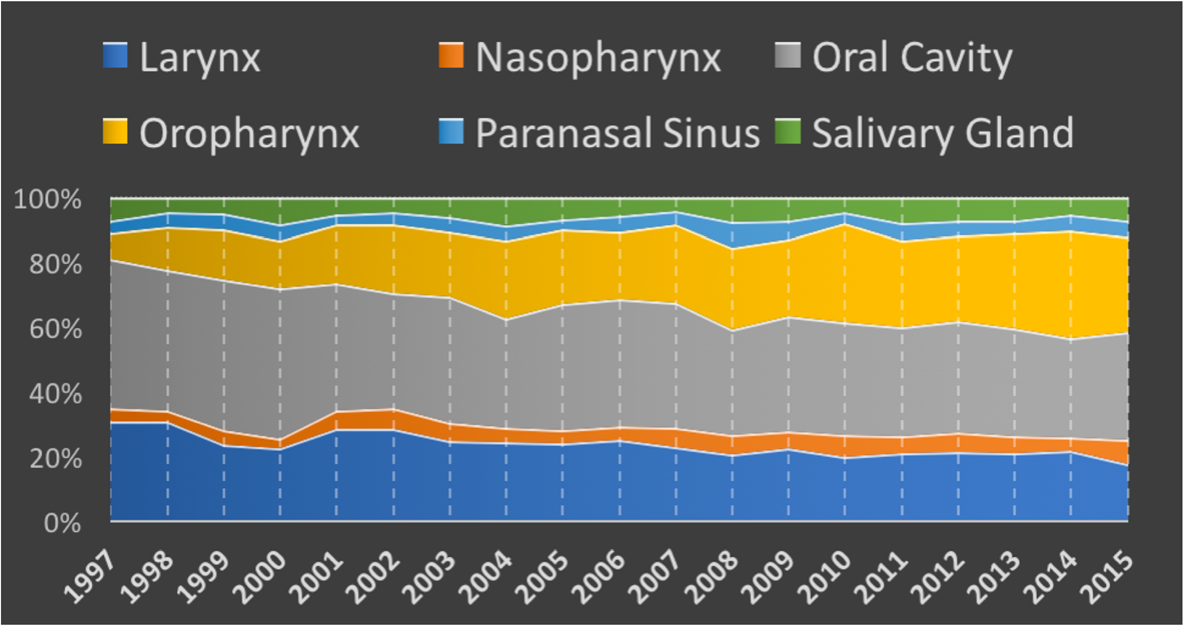
Percentage of New HNC Survivors by Subsite between 1997 and 2015

A total of 7562 HNC patients meeting inclusion criteria were identified in the ACR between 1997 to 2015 inclusive. During this time, the distribution of new HNC survivors (i.e. alive and one-year post-treatment) changed within Alberta (Fig. 1). In 1997, OCSCC represented 46% of new head and neck cancer survivors, while laryngeal cancer survivors made up 31% (Fig. 1). In 2015, new survivors of OCSCC and laryngeal cancers decreased to represent 33 and 18% respectively, while the proportion of new OPSCC survivors increased from 8% in 2005 to 29% in 2015 (Fig. 1). The proportion of nasopharyngeal, paranasal sinus, and salivary gland cancer survivors remained relatively stable over this time period (Fig. 1). In addition, the mean age at diagnosis for all HNC decreased significantly from 64 to 62 years of age (*p* = 0.04).

**Fig. 2:**
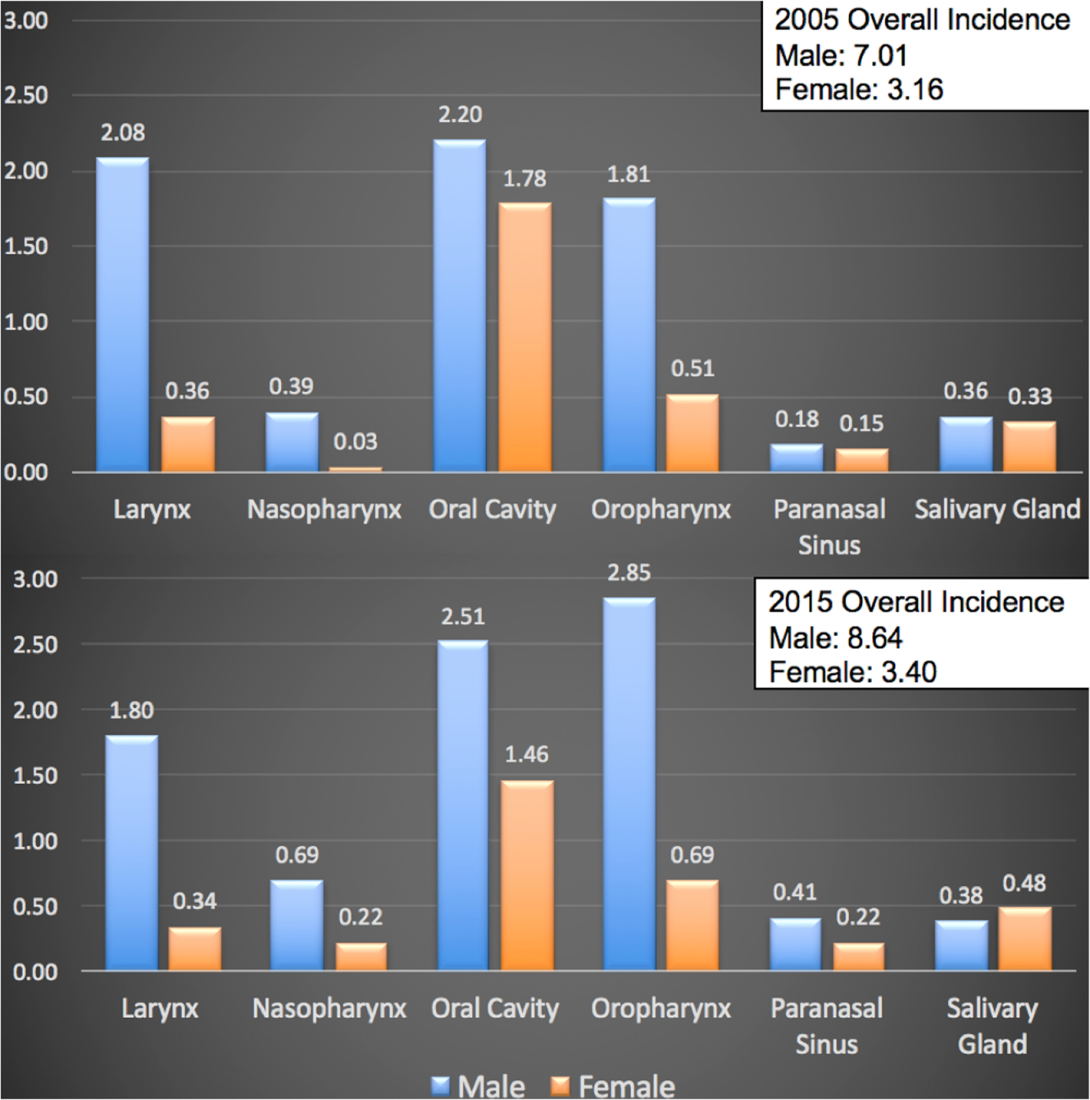
Incidence of New HNC Survivors by Subsite in 2005 and 2015 (cases per 100,000 people)

**Fig. 3:**
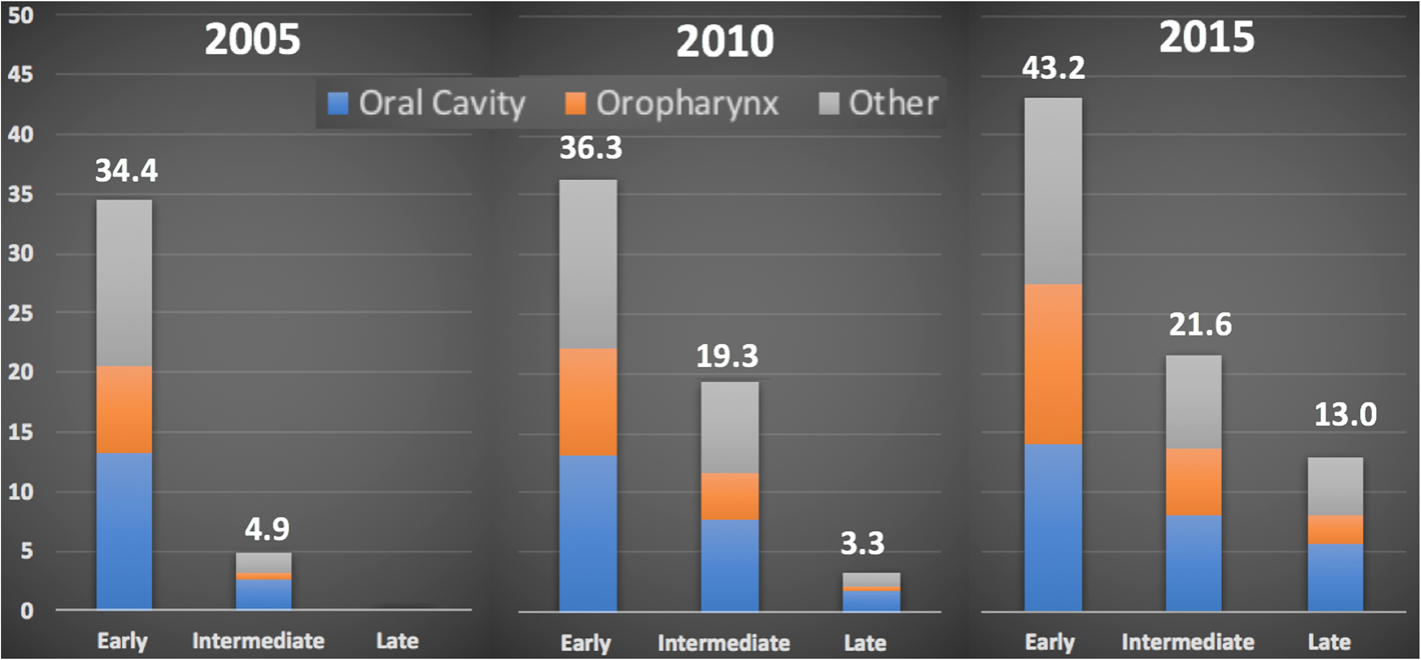
Point Prevalence of New HNC survivors by Subsite and Year (cases per 100,000 people)

Analysis of 6006 patients in the ACR between 2005 and 2015 demonstrated that the overall prevalence of HNC survivors nearly doubled from 39.3 to 77.8 per 100,000 (Fig. 3). The proportion of male HNC survivors increased from approximately 68.8 to 71.8%. The overall incidence of alive OPSCC survivors increased from 2.32 to 3.54 per 100,000 during this time period. The overall incidence of alive OCSCC survivors remained stable at 3.98 to 3.97 per 100,000 respectively (Fig. 2).

When comparing the overall point prevalence of HNC survivors between the three time periods, the overall prevalence per 100,000 in the “early” subgroup increased from 34.4 to 43.2 between 2005 and 2015 respectively (Fig. 3). In 2015, 13.0 HNC survivors per 100,000 were considered “late” survivors (i.e. at least 10 years post-treatment (Fig. 3).

## Discussion

To our knowledge, this is the first Canadian study to evaluate the epidemiological trends of HNC survivors stratified by early, intermediate, and late survivors. Our data estimates 78 new head and neck cancer survivors per 100,000 people in Alberta over a 15-year period. For a population of over 4 million people, this corresponds to over 3120 survivors added during this period. Of these, at least 520 are late survivors (i.e. more than 10 years post-treatment). The pattern of survivors mirrors what is known about new HNC incidence; namely, there are increasing numbers of younger, male oropharyngeal cancer survivors [2, 12, 13]. In fact, 71.8% of the head and neck cancer survivors were male, which is consistent with Canadian Cancer Society data [6]. Furthermore, concurrent with the decreasing rates of cigarette smoking [2], there is a smaller population of laryngeal cancer survivors [2]. However, oral cavity subsite still comprises a large proportion of survivors despite declining smoking prevalence in urban areas of Alberta since 2000 and alcohol consumption rates similar to Ontario [14].

Cancer survivorship issues are increasingly recognized as important topics by oncologic societies and this is particularly true for head and neck cancer [12, 13, 15]. The number of cancer survivors globally continues to rise due to population growth and aging as well as advances in diagnosis and treatment. In the United States, the number of Americans living with a history of cancer from 2016 to 2030 is projected to increase from 16.9 to 22.1 million respectively [6]. HNC cancer survivors are estimated to comprise 3% of all cancer survivors [16], with 15 and 41% of patients dying within 5 and 10 years respectively [13, 17]. With the demonstrated increase in overall and subsite specific survivorship in HNC patients, a paradigm shift towards rehabilitation to match longer periods of survivorship is already emerging. Prioritizing the survivorship experience will enhance our ability to provide quality patient centered care beyond the immediate post-treatment period. The emphasis on preserving functional outcomes was highlighted by Kim and colleagues who found that 25% of surveyed HNC survivors placed a greater emphasis on issues related to quality of life over curative status [18].

Our results corroborate the increasing proportion of HPV-associated OPSCC cases seen on a global scale. Our estimation of OPSCC cases comprising 29% of HNC diagnoses in 2015 is reflective of recent United States population studies approximating 31.2% in 2010 as outlined by Habbous et al. [1] The intersection of increasing incidence and favourable survival outcomes highlight the importance of acknowledging this growing cohort in future healthcare decisions.

While many HNC patients are discharged from follow-up by their oncologic care team after 5 years, our results demonstrate the increasing prevalence of intermediate and late subgroup survivors that represent a vulnerable and likely under-serviced population. The disparity in management of treatment associated morbidities is of particular concern [9]. Bozec and colleagues evaluated functional outcomes in OPSCC patients treated with primary surgical free flap reconstruction, and despite preserved functional and quality of life scales in over 70% of patients, complaints regarding oral intake, dentition, swallowing, xerostomia and trismus were common [8]. Of note, 36% of their study participants had mood disorders and approximately 52% returned to work [8], demonstrating the degree of psychosocial supports required.

There is also increasing awareness that organ preservation protocols of radiation and chemotherapy, particularly for laryngeal and oropharyngeal subsites, can lead to fibrotic changes that progress or worsen over time. Cohen et al. detailed radiotherapy effects when estimating esophageal stricture rates as well as shoulder dysfunction on 7 and 70% of HNC patients treated with radiotherapy respectively [16]. Studies examining late-effects (i.e. more than 10 years post-treatment) are still limited, but Hutcheson et al. have reported delayed dysphagia, strictures, and craniopathies in irradiated patients [19, 20]. These long-term are particularly important given the longer periods of survivorship expected for HPV-related oropharyngeal cancer patients [12, 16].

The Alberta Cancer Registry prospectively maintains information about new cancer cases and cancer-related deaths and is considered highly accurate and reliable [10]. This helps ensure low rates of missed data and minimizes bias seen in retrospective data collection. However, there are several limitations of our study. The disease status of patients in this study were not double-checked with either chart review or review of other electronic, provincial medical records. In addition, we did not stratify results by HPV status, although recent publications from our center illustrate the increasing proportion of HPV associated OPSCC [13]. This is mainly due to the fact that p16 status was not widely collected in the province until 2008. Finally, this study focused on survivors added to the population since 1997, as data regarding the baseline line number of head and neck survivors in the community prior to this was not available.

## Conclusion

Our study provides an evaluation of the changing epidemiological landscape of head and neck cancer survivors in Alberta and the broader catchment population of western Canada. Within our patient cohort, we see a rising proportion of OPSCC survivors reflective of global trends, with increasing prevalence of intermediate and late subgroup HNC survivors. As we can expect an increasing burden of survivorship in these patients, an emphasis on the evolving treatment associated morbidities and how to appropriately manage late complications will become forefront decision and research questions.

## Data Availability

Access to primary data requires ethics approval. Additional analyzed data not presented in the manuscript is available upon request.

## Abbreviations

HNC: head and neck cancer
HPV: Human papilloma-virus
OCSCC: oral cavity squamous cell carcinoma
OPSCC: oropharyngeal squamous cell carcinoma
ACR: Alberta cancer registry
